# Expanding HPV prevention across the life course: evidence on vaccine effectiveness in adults

**DOI:** 10.3389/fpubh.2026.1894760

**Published:** 2026-07-24

**Authors:** Lorena Bejarano-Hernández, Angela Chan, Meera Gosalia, Michael Moore, Bettina Borisch, Raman Bedi, Marta Lomazzi

**Affiliations:** 1 World Federation of Public Health Associations (WFPHA), Institute of Global Health, University of Geneva, Campus Biotech – G6, Geneva, Switzerland; 2 University College London Medical School, London, United Kingdom; 3 Health Research Institute, University of Canberra, Bruce, ACT, Australia; 4 Dental Institute, Kings College London, London, United Kingdom; 5 Institute of Global Health, University of Geneva, Campus Biotech – G6, Geneva, Switzerland

**Keywords:** adults, cervical cancer, HPV immunization, HPV vaccination, HPV vaccine, HPV-related cancers, human papillomavirus (HPV), vaccine effectiveness

## Abstract

Despite Human Papillomavirus (HPV) remaining a major global public health concern and affecting all age groups and genders, most HPV vaccination programmes mainly target female adolescents. This narrative review synthesizes and critically discusses recent evidence on the effectiveness of HPV vaccination among adults to inform its integration into adult immunization programmes. A substantial body of recent literature has examined the effectiveness of HPV vaccination in adult populations. Across the literature, vaccine effectiveness has been evaluated in relation to (1) HPV infection and viral dynamics, (2) precancerous cervical lesions (HSIL/CIN), (3) prevention of post-treatment recurrence, (4) HPV-related cancers, and (5) anogenital warts. Some associations were found between HPV vaccination and reduced HPV infection and viral persistence, whilst effect estimates varied widely across studies for high-grade cervical lesions (CIN2+). This review aims to characterize the effectiveness of the HPV vaccination amongst adults (18–45 years) in preventing human papillomavirus infections and HPV related disease outcomes. By synthesizing and evaluating recent evidence on HPV vaccine efficacy, we aim to assess its relevance in expanding HPV vaccination within life-course immunization programs.

## 1 Introduction

Human Papillomavirus (HPV) remains a major global public health concern due to its association with a range of cancers, including cervical, anal, oropharyngeal, vaginal, vulvar, and penile cancers (1–3). It is estimated that up to 80% of sexually active individuals will acquire the HPV infection at some point in their lives (4). Among HPV-related cancers, cervical cancer remains the most impactful in terms of mortality and health inequities, ranking as the fourth most common cancer among women globally, with approximately 350,000 deaths in 2022, 94% of which occurred in low- and middle-income countries (5). Of particular concern is the sustained increase in HPV-related oropharyngeal cancer (OPC), which now represents the most rapidly growing HPV-associated malignancy in high-income countries (6). Despite ongoing efforts, the global coverage rate remains slow, and the goal to eliminate cervical cancer as a public health problem by 2030 seems distant (7).

Historically, HPV prevention strategies have focused predominantly on female adolescents through school-based programs prior to sexual debut. While this has yielded population-level benefits (8), it has created a significant “catch-up” gap, resulting in large cohorts of adults and, particularly males, who remain unvaccinated due to age-based eligibility, gender, lack of access, or the absence of programs during their youth. Global coverage data show that 84% of women aged 30–49 years living in high-income countries had been screened throughout their life, compared with 48% in upper middle income-countries and 20% in lower middle income countries (9). This reflects a persistent gap in interventions targeting populations beyond female adolescents (10). When contextually appropriate and feasible, public health authorities are increasingly reframing immunization as a universal life-course intervention. This approach recognizes that everyone, not only young girls, remain at risk of HPV infection, can be reinfected and may benefit from protection against genotypes to which they have not yet been exposed thereby contributing to ongoing transmission and development of HPV-related cancers (11–13). Modelling evidence studies indicate that achieving cervical cancer elimination will require complementary strategies that address unvaccinated cohorts beyond adolescence (14).

While HPV vaccination programs should start with female and male adolescents, the rationale for expanding this focus to include adult women, men, and LGBTQ+ populations is grounded in both health equity considerations and immunological evidence (15). First, natural immunity following a previous infection does not confer complete protection against reinfection with other HPV genotypes; therefore, immunization can remain beneficial throughout adulthood. Furthermore, adult men represent a particularly underserved group, as they do not benefit from the screening programmes available to women and play an important role in sustaining HPV transmission dynamics (16, 17). In general, there are various barriers preventing adults from accessing the HPV vaccine, such as beliefs regarding necessity of vaccination for adults, vaccine safety, and concerns over individual cost (18). Extending HPV vaccination to these groups is now viewed as an essential element of wider cancer prevention and its economic benefits (12, 13).

Despite a growing consensus on the value of adult vaccination and the availability of HPV vaccines for use in adult populations uptake remains low largely due to structural, informational, societal, and policy-related barriers. These include insufficient awareness, stigma related to sexual health, misconceptions about the effectiveness of vaccines in older populations, associated costs, and the exclusion of adults from national immunization schedules (12, 13). This review examines recent evidence published between 2020 and 2025 on the impact of HPV vaccination in adults, with the aim of assessing recent evidence on its effectiveness within the broader framework of a life-course immunization strategy. Rather than systematically cataloguing studies, this paper aims to interpret emerging patterns and implications for policy and practice.

## 2 Methods

### 2.1 Criteria for study submission

The focus of the present review was on adults from the global population aged 18–45 years, who had received at least one dose of the HPV vaccination as an adult (i.e., aged 18 years and over) or were due to receive at least one dose of the HPV vaccination as part of the study. This included any adult who met these criteria regardless of sex, gender, sexual orientation (LGBTQ+ persons), and risk status for HPV infection.

Articles were eligible for inclusion if their primary aim was to investigate the effectiveness of the HPV vaccine. This included any articles which compared varying dosage levels of the HPV vaccines regardless of HPV vaccine type (bi-, qua-, or nonavalent). Articles were excluded if the HPV vaccine was used in combination with other drugs or vaccines.

Since the aim of the present review was to only review recent evidence of the effectiveness of the HPV vaccine and not to compare it to other vaccination types, the only acceptable comparator specified was groups who had never received the HPV vaccination including administration of placebo injections.

Studies were deemed eligible only if they reported evidence of the effectiveness of the HPV vaccine. Effectiveness was operationalized as HPV seropositivity rates, risk of infections, remission rates, or prevalence of high-grade squamous intraepithelial lesions (HSILs) and cervical intraepithelial neoplasia (CIN). This review aims to solely present evidence on the effectiveness of the vaccine from a population health perspective rather the effects of immunological mechanisms. Therefore, studies examining immunogenicity, antibody response or other such immune outcomes were not considered.

Studies which met the previously described criteria and explicitly assessing the efficacy of the HPV vaccine were considered for inclusion. These included observational studies, randomized controlled trials, and epidemiological and policy reports where appropriate. Case studies, commentaries, opinion letters, or reviews were not included.

### 2.2 Search strategy

This narrative review draws on recent literature published in 2020 up to and including the date of the search (3 October 2025). The focus on recent literature was intended to capture the most up-to-date evidence in the context of evolving vaccination strategies and newer vaccine formulations targeting adults. Relevant studies were identified through targeted searches across three databases. These databases were PubMed^1^, Scopus (Elsevier)^2^, and for grey literature, Google Scholar^3^.

The search terms or Medical Subject Headings (MeSH) terms entered into the database were guided by the specified inclusion and exclusion criteria for each criterion. A complete list of specific MeSH terms for each of the three databases are included in Appendix A.

The aim is not to provide an exhaustive synthesis but to interpret and contextualize emerging evidence across clinical and population-level outcomes. A summary of key included studies is provided in Supplementary Table S1 to support transparency and contextualization of the evidence discussed.

### 2.3 Screening

All records identified were first screened for duplicates. These duplicates were reviewed by both reviewers LB and AC, and removed. From this point, all screening procedures were done independently by reviewers LB and AC, each reviewer blind to the decisions made by the other. This was to ensure reliability of the screening process. Any conflicts in decisions made were resolved through discussion with the supervising researcher ML. Following removal of duplicates, all titles and abstracts were screened for relevance to the aim of the review. For records with eligible titles and abstracts, full texts were sought for retrieval, and records with available full texts were further screened for appropriateness. All relevant data pertaining to the outcome of interest was extracted from the publications that met all the criteria for inclusion.

## 3 Results

### 3.1 Evidence on HPV vaccine effectiveness in adults

Available evidence suggests that HPV vaccination in adults is associated with reduced prevalence of HPV infection and lower odds of HPV positivity even when vaccination occurs after sexual debut. Across several cohorts, vaccination was associated with substantial reductions in HPV infection and persistence among adults up to age 45 and, in some studies, up to age 60 (19–21). In HPV-infected Korean women aged 20–60 years with abnormal cytology, the adjusted prevalence ratio (adjPR) for HPV types 16/18 among vaccinated women compared with unvaccinated women was 0.51. Among women diagnosed with low-grade lesions (LSIL), those vaccinated more than 12 months earlier had a lower risk of HPV positivity (adjPR = 0.35) (20). Similarly, an Australian cohort study found significantly lower prevalence of the nine high-risk HPV types targeted by the nonavalent vaccine among vaccinated individuals compared with unvaccinated participants (0.9% vs. 3.4%, *p* = 0.022) (22). In Thailand, a cohort of women aged 20–45 years demonstrated an effectiveness of 84.6% in reducing HPV 16/18 positivity among vaccinated individuals (19). Evidence also suggested a reduction in persistent infection. In some cohort analyses in Korea, vaccination was associated with substantial reductions in persistent infection (e.g., around 50% in certain populations) (21). Of note, the timing of vaccination relative to sexual activity influences effectiveness. Since HPV vaccines are prophylactic and do not eliminate established infections, vaccination shortly after sexual debut appears to confer greater protection (23). For example, Canada reported substantially higher vaccine effectiveness among individuals who had been sexually active for 5 years or less before vaccination compared to those who had been sexually active beyond the 5 years. (PR = 0.15 for cumulative incidence) (24).

Vaccination in adults may also influence viral dynamics among individuals already infected. In Korea it was found that vaccination reduced HPV-16 viral load by approximately six-fold and shortened the duration of persistent infections (25). Similarly, in Poland, complete viral was observed in 72.4% of vaccinated women compared with 45.7% among unvaccinated women (26).

Evidence of effectiveness has also been observed among men who have sex with men (MSM). In the United States, vaccination was associated with lower prevalence of anal HPV infection among MSM aged 27–45, particularly when vaccination had been initiated at ages 18–26 or when at least 2 years had elapsed since the first dose (27).

Taken together, these findings suggest that HPV vaccination remains effective in reducing infection, persistence, and viral load even when administered in adulthood, although effectiveness is influenced by timing relative to exposure and prior infection status.

### 3.2 Impact on precancerous cervical lesions (HSIL/CIN)

Several studies demonstrate that HPV vaccination in adults contributes to reductions in high-grade cervical lesions. In China, women vaccinated between ages 20 and 45 demonstrated very high vaccine efficacy against HPV-16/18-related CIN2+ over a 13-year period (23). Similarly, in Korea, vaccinated women had a 62% lower risk of progression to CIN2 + compared with unvaccinated individuals (21). Evidence from Thailand also demonstrated a very high reduction in HPV-16/18-associated LSIL or higher-grade lesions over a median follow-up of 7.3 years among vaccinated women aged 20–45 (19). Population-based data from the Netherlands further suggest that vaccine-induced protection against high-grade lesions extends into adulthood. Fully vaccinated women showed a cumulative risk ratio of 0.19 for CIN3+ compared with unvaccinated women (28). Additional evidence from Japan reported an 86% reduction in CIN3+ amongst vaccinated individuals in nationwide analyses (29). Cohort analyses from Norway suggest that although effectiveness is highest when vaccination occurs before age 20, adult vaccination still provides protective benefits, though these are influenced by prior HPV exposure (30). A Canadian analysis found that British Columbia’s school-based HPV vaccination program was associated with reductions in CIN2 and CIN3 rates of 62% and 65%, respectively, among women aged 16–23 years (31).

Given the lower risk of high-grade lesions among vaccinated women, some studies have suggested that cervical screening protocols could potentially be adapted for vaccinated populations, including consideration of a later start age for screening.

Overall, the evidence reviewed here suggests that HPV vaccination in adults contributes to meaningful reductions in high-grade cervical lesions, although the magnitude of effect varies depending on age at vaccination and prior HPV exposure.

### 3.3 Prevention of recurrence after treatment

Emerging evidence suggests that HPV vaccination may also act as a secondary or tertiary prevention strategy among adults treated for HPV-related lesions. Among women treated for high-grade cervical lesions (CIN2-3), vaccinated individuals had significantly lower HPV positivity at follow-up compared with unvaccinated women (8% vs. 18%) (32). Similarly, in the Czech Republic post-excision vaccination, the nonvalent HPV vaccine was associated with a 90% reduction in the risk of CIN2+ recurrence (33). Norwegian findings further showed that vaccination following conization was associated with a 12.1% absolute reduction in HPV positivity at six-month follow-up, suggesting a lower risk of recurrence (34). Consistent results were reported in Spain, where vaccination was associated with a 57% reduction in persistence or recurrence of high-grade lesions post surgical treatment (35).

Collectively, these findings support a potential role for HPV vaccination as an adjunct strategy in reducing recurrence after treatment, highlighting its relevance not only for primary but also for secondary and tertiary prevention.

### 3.4 Impact on HPV-related cancers

Evidence from the included population-level studies suggest that reductions in HPV infection and precancerous lesions are translating into reductions in HPV-related cancers. In the Netherlands, fully vaccinated women demonstrated an 81% reduction in CIN3+ and a 91.5% reduction in cervical cancer risk compared with unvaccinated women (28). Analyses from the U. S. Surveillance, Epidemiology, and End Results (SEER) program also showed that while adenocarcinoma incidence historically increased, rates stabilized or declined among age groups eligible for HPV vaccination (20–29 years), whereas they continued to increase in older, unvaccinated populations (36). Data from the U.S. National Health and Nutrition Examination Survey (NHANES) further suggest that the prevalence of ever-diagnosed cervical cancer was substantially lower among vaccinated women (0.46%) compared with unvaccinated participants (1.27%) (37). There is also an impact on oropharyngeal cancers, with a 19-fold higher risk of developing oropharyngeal cancer in unvaccinated compared with vaccinated individuals; among men, the risk was approximately 23.8 times higher (38).

Although evidence on cancerous outcomes remains limited, the available data suggest that reductions in infection and precursor lesions are translating into measurable decreases in HPV-related cancers, reinforcing the long-term benefits of vaccination beyond adolescence.

### 3.5 Impact on anogenital warts

HPV vaccination is also associated with reductions in the incidence of anogenital warts. Data from national immunization programs demonstrate that vaccination substantially lowers the burden of genital warts at the population level. In Germany, a retrospective analysis of women aged 28–33 years reported a 38.6% decline in anogenital wart prevalence following introduction of the HPV immunization program (39). Vaccination may also reduce recurrence after treatment. Adjuvant HPV vaccination has been associated with lower recurrence of condyloma acuminata following surgical removal (26). Preliminary evidence also suggests potential benefits for preventing infection, reinfection, or post-treatment recurrence. In adults with treatment-resistant palmoplantar warts, vaccination resulted in complete remission in 11% and partial remission in 39% of participants after 12 months (40). However, effectiveness may vary depending on underlying health status, with evidence showing that vaccination reduced acquisition of HPV type 6 but did not significantly reduce the incidence of anogenital lesions compared with placebo in HIV-positive MSM (41).

## 4 Discussion

### 4.1 Summary of main findings

This review examined the existing evidence from recent years (2020 to 2025) on the effectiveness of the HPV vaccine among adults aged 18 and older. Across the representative literature reviewed here, the HPV vaccine was assessed on five identified outcomes: (1) HPV infection prevalence, (2) (precancerous) HSILs and CINs, (3) recurrence prevention post-treatment, (4) HPV-related cancer prevalence, and (5) anogenital warts. Most of the included studies focused on adult populations aged approximately 26 to 40–49 years, with more limited evidence available for individuals beyond 45 years of age. This likely reflects historical vaccination eligibility as well as the relative scarcity of data in older adult populations, highlighting an important gap in the literature.

Firstly, populations vaccinated against HPV were up to half as likely as their unvaccinated counterparts to test positive for HPV infection. Evidence suggests a sustained protective effect against HPV infection lasting up to 13 years, alongside reductions in viral load of up to six-fold among individuals with persistent infection (25). However, other studies highlight greater effectiveness when vaccination occurs closer to sexual debut particularly by no more than 5 years, suggesting that vaccination at a younger age may be associated with improved effectiveness (24). Nonetheless, evidence of the effectiveness of the HPV vaccine against HPV infection in adults is present in the literature, including in MSM for whom the vaccine has been found to significantly reduce the incidence of anal HPV 6/11/16/18. This is consistent with an earlier systematic review and meta-analysis by Ellingson et al. (42) who found that while greater antibody response following HPV vaccination is observed among younger adolescents, the response observed among those in later adolescence and adulthood also results in considerable immunity against HPV infection. Importantly, heterogeneity across studies reflects differences in population characteristics. Evidence suggests that vaccine effectiveness is generally higher when administered at younger ages, particularly closer to sexual debut, although meaningful benefits are still observed in older adults. Differences by sex are also relevant, with increasing evidence supporting effectiveness among males, including men who have sex with men, a group at higher risk of HPV-related disease. In addition, outcomes may vary across risk populations, with some evidence suggesting reduced effectiveness in immunocompromised individuals. These variations highlight the importance of considering subgroup-specific strategies when interpreting the evidence and designing vaccination policies. However, given the narrative nature of this review, the differences between these subgroups are interpreted qualitatively rather than through formal stratified analysis.

With respect to precancerous lesions, while variability exists across studies, the overall trend supports effectiveness of the HPV vaccine against HSILs and CINs with some studies finding significant associations between HPV vaccination and a reduction in referrals for CIN2+, progression of lesions to CIN2+, and recurrence of CINs, as well as lower risk of CIN3+ for partial vaccinations. Some caveats to this effectiveness, however, included immunosuppressed status and non-metropolitan statistical areas (non-MSAs), older age at vaccination receipt, for which HPV vaccination was not significantly associated with lower risk or prevalence of CIN2+. Four other studies also did not find any significant effectiveness of the HPV vaccination on domains such as CIN2+ diagnosis, relapse, and reinfection. This is in line with a systematic review by Gonçalves et al. (43) which found clinical efficacy of the vaccine for CIN2/3. As highlighted by the authors, however, the evidence base for the HPV vaccines in the treatment of CINs remains sparse.

Of the publications reporting the effect of HPV vaccine on HPV-related anogenital warts, only one yielded explicitly significant findings: the HPV vaccine was linked to a significant decline in the prevalence of anogenital warts (39). This is, nonetheless, consistent with an earlier meta-analysis of eight randomized controlled trials published from 2006 to 2019 which found that the HPV vaccine significantly reduced incidence and prevalence of genital warts in both young women and men (44). Despite the small number of studies in this review, considering them alongside previous findings in the literature suggests the HPV vaccine is effective at reducing the prevalence of anogenital warts. At the same time, however, as noted by the authors, the rates of complete and partial remission reported by Merio et al. (40) are lower than earlier studies have previously reported [e.g., 56, 57], which they attribute to the older age, immunocompromised status, and greater severity of the warts among their sample. Some reports found no significant difference in the rate of onset of anogenital lesions between HIV-positive MSM with and without the HPV vaccine. The consistency between these results and findings by Silberberg et al. (55) citing the ineffectiveness of the HPV vaccine at preventing CINs in immunosuppressed patients warrants further inquiry into the prophylactic effect of the HPV vaccine in immunosuppressed patients. Furthermore, because current data on immunosuppression is predominantly from populations with HIV, other immunodeficiency disorders require further investigation.

Despite only few studies reporting the effect of the HPV vaccine on cancer incidence and risk, the available evidence suggests that the vaccine is effective at reducing the risk of HPV-related cancers, specifically cervical cancer, adenocarcinoma, and oropharyngeal cancer. Importantly, HPV vaccination was associated with substantial reductions in the risk of OPC in men which provides strong empirical support for the case for including male adults in HPV vaccination efforts.

Altogether, the four outcomes identified here appear congruent with an earlier systematic literature review which also found the HPV vaccine to be effective on three related outcomes: reduction of infection, precursor lesions, and cervical cancer (45). While HPV vaccination may be most effective for receipt prior to sexual debut, this review presents evidence that also suggests a protective effect and potential role in reducing recurrence across various clinical outcomes when administered further into adulthood. Taking the evidence summarised here provides a strong basis for the consideration of HPV vaccination as a life course intervention. In this context, prioritization strategies adapted to different country settings and health system realities, such as targeted catch-up vaccination for younger adults and high-risk groups first, may help maximize both individual and public health benefits. Importantly, these findings challenge the traditional assumption that HPV vaccination is primarily beneficial only before sexual debut.

While this review has focused on HPV vaccine effectiveness, it is worth noting that evidence suggests expanding vaccination to older ages or specific at-risk populations can also generate meaningful economic returns. Modelling data further show that catch-up vaccination through age 34 years approaches cost-effectiveness, and that broader expansion would prevent tens of thousands of additional cancers and deaths over a 100-year horizon. Although further inquiry is needed, this suggests that even modest program expansions carry a measurable return on investment when measuring prevention of cancers, pre-cancerous lesions, and genital warts (46, 47).

### 4.2 Limitations and implications for future research and policy practice

The primary limitation of this narrative synthesis relates to the selection of studies included for analysis. Restricting the search to articles published from 2020 to 2025 was intended to capture the most current evidence relevant to the evolving paradigm of life course immunisation (i.e., extending vaccine strategies to adults). However, limiting the temporal scope of the search severely limits the breadth of evidence reviewed here. As such, the evidence we review is by no means an exhaustive review of all available evidence on the effectiveness of the HPV vaccine in adults.

The current evidence base is limited by the underrepresentation of less developed regions. As evidenced by de Martel et al. (48), HPV-attributable cancers of the head and neck are most prevalent in North America and Northern Europe, while cervical cancers are observed more frequently in less developed regions, particularly Southeast Asia, South America, and sub-Saharan Africa. Across the 60 studies reviewed here, only two underdeveloped countries were represented: India and Thailand. This reflects a general gap in evidence synthesis off HPV vaccine efficacy. The unequal evidence base can be broadly attributed to the geographical burden of cervical cancer, especially in India and China, who account for nearly 40% of the global cervical cancer rate, and therefore should take precedence in any life-course HPV vaccination strategy (2, 5). China contributed very few studies to the present review (23, 50, 51, 53), and several of these demonstrated high and sustained vaccine effectiveness over extended follow-up periods. Similarly, limited evidence was produced from India (54), reflecting the historically low rates of HPV vaccine coverage in the country despite its substantial cancer burden.

However, global estimates from a pooled analysis of data from 2006 to 2014 revealed that while 118 million women have been targeted in HPV immunisation strategies, women from low- and lower-middle-income regions comprised only 1% of this number (49). While this may account for the lack of studies on populations from these regions, the inherently low rate of vaccine coverage in these regions and low vaccine effectiveness in non-MSAs (52)equally emphasise the need for vaccination programmes in these regions, as well as involvement of these regions in future studies to assess the effectiveness of the HPV vaccine in broader geographical and socioeconomic contexts. Most studies reviewed used entirely female samples which reflects the disproportionate focus of males in HPV immunisation programmes. Another limitation of this review pertains to the quality and heterogeneity of the included evidence. Indeed, the overall quality of the evidence varies, and several studies present potential sources of bias, including missing data, inappropriate analytical methods, and selective reporting of outcomes. Notably, some studies did not report measures of statistical significance (e.g., *p*-values or confidence intervals), which also limits the interpretation of findings. In addition, differences in statistical power across studies, often driven by sample size and study design, may partly explain variability in findings and should be considered when interpreting the evidence reviewed here. Lastly, studies reporting no benefit or potential negative associations with HPV vaccination may be less likely to be published, resulting in a possible overrepresentation of positive findings in the available evidence. Therefore, the results should be interpreted with caution.

Nevertheless, the present findings, congruent with previous work in this subject area, serve as a basic insight into the effectiveness of HPV vaccination in adults, which should be used to inform policies regarding HPV vaccine implementation for adults. Additionally, by identifying the five predominant domains on which HPV vaccine effectiveness can be assessed, this review provides a basic direction for future research.

## 5 Conclusion

The evidence reviewed in this narrative synthesis provides a strong basis for HPV vaccination beyond adolescence. Across adult populations, vaccination was consistently associated with reductions in HPV infection, viral persistence, high grade precancerous lesions, recurrence following treatment and HPV-related cancers and anogenital warts. Despite greatest efficacy present before sexual debut, these findings demonstrate substantial protection can still be achieved if vaccination is administered in adulthood, including among sexually active individuals and populations at elevated risk of HPV-related disease.

These findings support HPV vaccination as a broader life-course immunisation programme rather than limiting it to adolescence. In particular, the evidence highlights potential opportunities for catch-up vaccination strategies, post-treatment vaccination, and expanded access for populations historically underserved by HPV prevention efforts, including adult men and other at-risk groups.

Important evidence gaps persist, particularly in lower- and middle-income countries, among older adults, and in immunocompromised populations. Future high-quality longitudinal studies are needed to strengthen estimates of effectiveness across diverse settings and to better align the evidence base with the global distribution of HPV-related disease burden. Nevertheless, overall direction of the evidence remains consistent with HPV vaccination in adulthood contributing to reduced burden of HPV-related disease and should be considered as part of comprehensive and sustainable life-course immunisation strategies to accelerate progress towards reducing HPV-related disease worldwide.

## Footnotes

1
https://pubmed.ncbi.nlm.nih.gov/

2
https://www.elsevier.com/en-gb/products/scopus

3
https://scholar.google.com

## Glossary

HPV: Human papillomavirus
CIN: Cervical intraepithelial neoplasia
CIN2+: Cervical intraepithelial neoplasia grade 2 or worse
CIN3+: Cervical intraepithelial neoplasia grade 3 or worse
HSIL: High-grade squamous intraepithelial lesion
LSIL: Low-grade squamous intraepithelial lesion
ASC-US: Atypical squamous cells of undetermined significance
ASC-H: Atypical squamous cells, cannot exclude HSIL
MSM: Men who have sex with men
PRISMA: Preferred reporting items for systematic reviews and meta-analyses
OR: Odds ratio
HR: Hazard ratio
RR: Risk ratio
IRR: Incidence rate ratio
PR: Prevalence ratio
adjPR: Adjusted prevalence ratio
aOR: Adjusted odds ratio
aHR: Adjusted hazard ratio
aIRR: Adjusted incidence rate ratio
CI: Confidence interval
VE: Vaccine effectiveness
CRR: Cumulative risk ratio
AAPC: Average annual percentage change
SEER: Surveillance, epidemiology, and end results
NHANES: National health and nutrition examination survey
OPC: Oropharyngeal cancer
ICC: Invasive cervical cancer
LEEP: Loop electrosurgical excision procedure
MSA: Metropolitan statistical area
HDI: Human development index
9vHPV: 9-valent human papillomavirus vaccine
