# Supplementary material for: Expanding HPV prevention across the life course: evidence on vaccine effectiveness in adults
Source: Front Public Health. 2026 Jul 24;14:1894760. doi: 10.3389/fpubh.2026.1894760 (PMC13447305; doi:10.3389/fpubh.2026.1894760)
Supplement: Supplementary file 1 [file Supplementary_file_1.DOCX]

Supplementary Material

# 1 Supplementary Data

Appendix A. MeSH terms by database

1. PUBMED

("Adult"[MeSH] OR "Middle Aged"[MeSH] OR adult*[tiab] OR "middle aged"[tiab] OR "Young Adult"[MeSH] OR "young adult*"[tiab] ) NOT ( "Child"[MeSH] OR "Infant"[MeSH] OR "Adolescent"[MeSH] OR child*[tiab] OR infant*[tiab] OR adolescent*[tiab] OR teen*[tiab] OR teenager*[tiab] OR pediatr*[tiab] ) AND ( "Papillomavirus Vaccines"[MeSH] OR "Papillomavirus Infections"[MeSH] OR "Human papillomavirus"[tiab] OR "human papilloma virus"[tiab] OR HPV[tiab] ) AND ("Vaccination"[MeSH] OR vaccin*[tiab] OR immuniz*[tiab] OR immunis*[tiab] ).

2. SCOPUS

( TITLE-ABS-KEY ( adult* ) OR TITLE-ABS-KEY ( "middle aged" ) OR TITLE-ABS-KEY ( "young adult*" ) OR TITLE-ABS-KEY ( aged ) ) AND ( TITLE-ABS-KEY ( "papillomavirus vaccines" ) OR TITLE-ABS-KEY ( "HPV vaccine*" ) ) AND NOT ( TITLE-ABS-KEY ( child* ) OR TITLE-ABS-KEY ( infant* ) OR TITLE-ABS-KEY ( adolescent* ) OR TITLE-ABS-KEY ( teen* ) OR TITLE-ABS-KEY ( teenager* ) OR TITLE-ABS-KEY ( pediatr* ) ) AND ( LIMIT-TO ( EXACTKEYWORD,"Human" ) OR LIMIT-TO ( EXACTKEYWORD,"Adult" ) OR LIMIT-TO ( EXACTKEYWORD,"Female" ) OR LIMIT-TO ( EXACTKEYWORD,"Humans" ) OR LIMIT-TO ( EXACTKEYWORD,"Papillomavirus Infection" ) OR LIMIT-TO ( EXACTKEYWORD,"Vaccination" ) OR LIMIT-TO ( EXACTKEYWORD,"Middle Aged" ) OR LIMIT-TO ( EXACTKEYWORD,"Human Papilloma Virus Vaccine" ) OR LIMIT-TO ( EXACTKEYWORD,"Young Adult" ) OR LIMIT-TO ( EXACTKEYWORD,"Human Papillomavirus" ) OR LIMIT-TO ( EXACTKEYWORD,"Hpv" ) OR LIMIT-TO ( EXACTKEYWORD,"Hpv Vaccine" ) OR LIMIT-TO ( EXACTKEYWORD,"Immunization" ) OR LIMIT-TO ( EXACTKEYWORD,"Hpv Vaccination" ) OR LIMIT-TO ( EXACTKEYWORD,"Public Health" ) OR LIMIT-TO ( EXACTKEYWORD,"Human Papillomavirus Vaccine" ) OR LIMIT-TO ( EXACTKEYWORD,"Human Papilloma Virus" ) )

3. GOOGLE SEARCH

"human papillomavirus" OR HPV) vaccine adults -child -adolescent -infant -teen since:2020

# 2 Supplementary Table

**Table S1. Evidence of vaccine effect and outcomes in selected articles**

| Nº | Author (Year) | Country | Outcome | Vaccine Effect |
| --- | --- | --- | --- | --- |
| 1 | Abbas et al. (2023) | Germany | HPV infection Prevalence | 23% of HR-HPV types were not covered by the nonvalent vaccine (HPV 35/39/51/56/59). Most common HR-HPV types: 16, 56, 52, 51. Overall HPV prevalence 10.6% among unvaccinated women aged >30. |
| 2 | Abel et al. (2021) | England | HPV infection Prevalence | Prevalence of 6, 11, 16 and 18 infections were lower in vaccinated individuals with:    1 dose (0.3%, 95% CI=0.0% to 0.9%)  2-3 doses (0.4%, 95% CI=0.0% to 1.2%)    And unvaccinated individuals:    (1.2%, 95% CI=0.9% to 1.6%) |
| 3 | Abi-Raad et al. (2025) | United States | Precancerous Cervical Lesions    CIN2 | HPV non 16/18 more prevalent than 16/18 in high-grade lesions. ASC-H: 68% non-16/18 vs HSIL:50% (p=.003).    CIN2+ cases: 56%non 16/18 positive (p=.04).    Trend to higher non-16/18 in vaccinated vs unvaccinated women; not statistically significant |
| 4 | Bennis et al. (2024) | United States | HPV infection Prevalence | HIV-negative MSM (n=82).  Vaccinated men (35.6%) had L1 HPV antibodies (93.1%) and lower prevalence of active anal HPV infection (20.7%) compared with unvaccinated men (32.1%). |
| 5 | Brouwer et al. (2022) | England | HPV infection Prevalence | Vaccination associated with reduced cervicogenital HPV infection incidence (HR 0.63; 95%CI 0.47-0.83) but not oral HPV infection.    Oral HPV infections highly transient (mean clearance 46 days) vs cervicogenital (87 days); only 16% of oral genotypes persisted vs 56% cervicogenital |
| 6 | Casajuana-Pérez et al. (2022) | Spain | Precancerous Cervical Lesions    CIN2  CIN3 | Persistent/recurrent HSIL/CIN 2-3 was presented by 4.3% vaccinated women and 9.8% in the non-vaccinated women (HR: 0.43, 95% CI: 0.22–0.84, p=0.014).    Vaccination was associated with the 57% reduction in HSIL persistence/recurrence after treatment. |
| 7 | Chambers et al. (2023) | Canada | HPV infection Prevalence | Participants who received their first dose at age ≤23 years and 0.15    Incidence: PRs:0.23 (95% CI, .05–1.03)  Persistence: 0.08 (95% CI, .01–.59)    Participants who were sexually active for ≤5 years before vaccination.  Incidence: PRs: 0.15 (95% CI, .03–.68)  Persistance: PRs: 0.12 (95% CI, .03–.54) |
| 8 | Chen et al. (2023) | China | Precancerous Cervical Lesions    CIN2 | HPV infection rates significantly lower in vaccinated group at year 1 (p=.049) and year 2 (p=.026). CIN2+ recurrence: Vaccinated 2.03% vs unvaccinated 10.62%. Unvaccinated had higher recurrence risk (OR=12.35, 95%CI 1.919-79.49, P.008) Reduction: 81% |
| 9 | del Pino et al. (2020) | Spain | Precancerous Cervical Lesions    HSIL | Post-conization cohort (V=153, NV112).  Persistent/recurrent HSIL: V 3.3% vs NV 10.7% (P=.015).  Vaccination associated with reduced HSIL recurrence (OR 0.2, 95%CI 0.1-0.7, P=.010) |
| 10 | DeSisto et al. (2025) | United States | HPV infection Prevalence | MSM without HIV (N=6,350)    Ages 18-26 :  vaccinated <18y aPR 0.13 (95%CI 0.08-0.22, VE=87%)  vaccinated >2y prior aPR 0.52 95%CI 0.42-0.64, VE=48%)    Ages 27-45 :  vaccinated at 18-26y aPR 0.68 (95%CI 0.57-0.82, VE=32%)  vaccinated >2y prior aPR 0.66 (95%CI 0.57-0.77, VE=33%) |
| 11 | de Souza et al. (2023) | Australia | HPV infection Prevalence | Oral HPV prevalence 7.2%; 53% carried HR-HPV types (no difference by vaccination status, p=.979).    Nonvalent vaccine-type oral HPV prevalence significantly lower in vaccinated vs unvaccinated (0.9% vs 3.4%, p=.022) |
| 12 | Donken et al. (2021) | Canada | Precancerous Cervical Lesions    CIN2  CIN3 | Reduction postvaccination in women 16-23 years  CIN2: 62% (95% CI 54-68%)  CIN3: 65% (95% CI 58-71%)  Women 24-28 years: No significant decline to CIN2 and CIN3 rates over the same period |
| 13 | Dong et al. (2023) | Norway | Precancerous Cervical Lesions    CIN2 | CIN2+ incidence per 100.000 at ages 25-29 : unvaccinated 637, vaccinated <20y 487, vaccinated >20y 831.    Adjusted IRR : vaccinated <20y aIRR 0.62 (95%CI 0.46-0.84) : vaccinated >20y aIRR 1.22 (95%CI 1.03-1.43) |
| 14 | Dvořák et al. (2024) | Czech Republic | Precancerous Cervical Lesions    CIN2 | CIN2+ recurrence per 100.000 person-days: unvaccinated 18 vs vaccinated 2[per 100.000 person-days]. 90% reduction in CIN2* recurrence (95%CI 12-99%), mean follow-up 30 months |
| 15 | Fappani et al. (2021) | Italy | HPV infection Prevalence | Italian adults aged 18–40 (n=393).  Overall HPV prevalence 19%; higher in females than males (22.1% vs 13.6%, p = .037).  Vaccine-type prevalence reduced in vaccine-eligible female cohort 10 years post-program. Frequency of vaccine types increased with age, particularly in males in pre-screening and screening cohorts (p = .029). |
| 16 | Gheit et al. (2023) | India | HPV-related cancers | Indian women, sexually active (n=997; V=818, UV=179).  Oral HPV16/18: vaccinated ASP 2.0% (95%CI 1.0–3.0%) vs unvaccinated 4.2% (95%CI 1.2–7.2%).  HPV16 by dose: 1-dose 3.5%, 2-dose 1.2%, 3-dose 1.5%, unvaccinated 3.3%. |
| 17 | Gilham & Peto (2025) | England | Precancerous Cervical Lesions | Post-HPV vaccination (girls born since 1991): cervical cancer incidence fell 90% in women <25y and 80% at ages 25–29 (2015–2022).  Pre-vaccination: raising screening start age from 20 to 25 doubled incidence at ages 25–29 and increased mortality by 77% (2000–2004 to 2010–2014). |
| 18 | Goyani et al. (2020) | New Zealand | Precancerous Cervical Lesions    CIN2  CIN3 | Post-vaccination trends (2011/2013–2017):  Auckland: 38% decrease in referrals, 55% decrease in CIN2+.  Waikato: 8% decrease in referrals, 22% decrease in CIN2+.  Canterbury: 24% decrease in referrals, 49% decrease in CIN2+.  Non-Māori CIN3+ proportion declined (24% to 16%, p < .001); no change in Māori women (31% to 29%, p = .90). |
| 19 | Graça & Brollo (2024) | Brazil | Preventing recurrence after treatment | Prevalence ratios of HPV-induced cervical lesions (relative to 2020).  Brazil overall PR 0.85,  North PR 0.75,  Southeast PR 0.83,  South PR 0.83 (all in 2023).  No significant reductions in Northeast or Central-West regions. |
| 20 | Guiqian et al. (2020) | China | HPV infection Prevalence | Overall HPV prevalence pre- vs post-vaccine introduction: 17.74% vs 17.11% (χ²=1.920, p > .05, not significant).  Most common genotypes: HPV52, HPV16, HPV58, HPV39. |
| 21 | Goodman et al. (2024) | Germany | HPV infection Prevalence    Precancerous Cervical Lesions    HPV-related cancers    Anogenital warts | Pre- vs post-NIP prevalence: CIN2+ declined 51.1% (0.92% to 0.45%, OR 0.49).  Anogenital warts declined 38.6% (0.44% to 0.27%, OR 0.60).  Vaginal precancer/cancer declined 75.0% (0.04% to 0.01%).  Anal precancer/cancer: too few cases for analysis. |
| 22 | Hidalgo-Tenorio (2021) | Spain | Anal HSIL | No significant difference in anal HSIL (V 14.1% vs placebo 13.1%, p = .98) or EAGL (V 11.1% vs placebo 6.8%, p = .4).  Protective effect against HPV6 at 12 months (V 7.5% vs placebo 23.4%, p = .047), not sustained.  Antibody response significantly higher in vaccinated arm at all timepoints (p = .0001).  Protective factor against HSIL: completing vaccination >6 months prior (OR 0.869, 95%CI 0.825–0.917). |
| 23 | Hu et al. (2022) | Costa Rica | HPV infection prevalence          CIN2+ prevalence    CIN3+ prevalence | HPV16/18/31/33/45 predominant (69%) among unvaccinated participants  HPV35/52/58/39/51/56/59/66/68 predominated (65%) among vaccinated participants.    Unvaccinated = 4.5% vs Vaccinated = 2.6% (*p* < .001)    Unvaccinated = 2.3% vs Vaccinated = 1.8% (*p* = .170) |
| 24 | Ikeda et al. (2021) | Japan | Precancerous Cervical Lesions      Abnormal cytology    CIN1+    CIN2+    CIN3+ | Vaccinated vs unvaccinated  OR0.42 (95%CI = 0.34 - 0.50)  OR 0.42 (95% CI, 0.31- 0.58),    OR 0.25 (95% CI, 0.12-0.54),    OR 0.19 (95% CI, 0.03-1.15), |
| 25 | Ikeda et al. (2025) | Japan | Precancerous Cervical Lesions      CIN2+    CIN3+ | Vaccinated vs unvaccinated  OR0.23 (95%CI=0.12-0.46)    OR0.14 (95%CI 0.03-0.75) |
| 26 | Issanov et al. (2022) | United States | HPV-related cancer | Vaccinated vs unvaccinated  adjOR 0.58 (95% CI = 0.19 – 1.75) |
| 27 | Ju et al. (2025) | Australia | HPV-related cancer      Oral HPV infection | Unvaccinated vs vaccinated  OR 1.08 (95% CI = 1.00–3.11) |
| 28 | Katz (2021) | United States | HPV infection prevalence | RR 19.37, 95% CI 7.27  to 51.62, P = .0001) (NV vs V risk); 23.8 for males *(p* = .0015); females 9.34 (*p* = .0001) |
| 29 | Kudo et al. (2022) | Japan | Precancerous Cervical Lesions    HPV 16/18 positivity    HSIL | Vaccinated vs unvaccinated  OR0.04 (95%CI = 0.01 – 0.18)    OR 0.22 (95%CI= 0.05 – 0.89) |
| 30 | Kassam (2025) | Canada | HPV infection prevalence | Individual-level: no association with HPV 6/11/16/18 prevalence (aOR 1.12, 95%CI 0.56–2.22) or incidence (aHR 0.34, 95%CI 2.19×10⁻¹⁸–1.38).  HPV-level: no prevalence association (aOR 0.99, 95%CI 0.57–1.71), but significant reduction in HPV 6/11/16/18 incidence (aHR 0.22, 95%CI 6.01×10⁻¹⁸–0.79). |
| 31 | Kitamura (2023) | Japan | HPV infection prevalence | HPV-16/18 positivity: 5.6% overall.  Highest vaccination rate in ages 20–24 (60.6%); lowest HPV-16/18 positivity in ages 45–49 (2.8%) and 20–24 (4.0%).  Vaccination significantly reduced HPV-16/18 infection risk (aOR 0.047, 95%CI 0.011–0.196). |
| 32 | Kurosawa (2022) | Japan | HPV 16/18 prevalence      HPV 31/45/52 prevalence      High-risk HPV types | Vaccinated = 0% vs unvaccinated =5.4% (*p* = .0018)  Vaccine effectiveness 100%    Vaccinated = 3.3% vs unvaccinated = 10.0%  (*p* = .013)    Vaccinated = 21.3% vs unvaccinated =23.7%  *(p = .*63) |
| 33 | Li et al. (2023) | United States | HPV-related cancer | 58% overall (aPR 0.42); by age: 64% (18–22y),  65% (23–27y).  Cross-protection in 18–22y vaccinated before sexual debut:  VE 47% against 13 non-vaccines  HR-HPV types  VE 61% (95%CI 36–77%) against HPV 35/39/52/58/59. |
| 34 | Lin et al. (2023) | Taiwan | HPV infection prevalence | Predominant HR-HPV:  HPV52, 58, 16 (>70% of cases annually).  Over the decade, significant declines in HPV31, 33, 39, and 51.  HPV31 and 33 are covered by 9vHPV vaccine;  HPV39 and 51 declines suggest possible cross-protection. |
| 35 | Lee et al (2022) | Thailand | HPV infection prevalence | HPV16/18 cases: V=0 NV=4;      84.6% reduction (95%CI = 43.5 - 95.8)    Reduction HPV16/18 ASC-US+ (effectiveness 88.0%; 95%CI 2.0 to 98.5) |
| 36 | Loenenbach (2023) | Germany | HPV infection prevalence | HPV16/18 46.4% (95%CI 4.2 - 70.01)    Any genotype 49.1% (95%CI 8.2 - 71.8) |
| 37 | Lonky et al (2021) | United States | Precancerous Cervical Lesions    CIN2+ | CIN2+ diagnosed in 23.2%; 40% were HPV16/18+.  No association between vaccination and CIN2+ diagnosis:  overall aOR 1.07 (95%CI 0.70–1.64);  pre-sexual debut aOR 1.11 (95%CI 0.55–2.24);  vaccinated <18y aOR 0.96 (95%CI 0.49–1.91);  complete dosing aOR 0.84 (95%CI 0.53–1.35).  No association with HPV16/18+ CIN2+ (p > .45). |
| 38 | Markowitz (2020) | United States | HPV infection prevalence | aPR 0.06 (V ≤18y)    Women vaccinated aged >18y:  4vHPVtype prevalence lower among unvaccinated women but not significant |
| 39 | Merio et al. (2023) | France | Preventing recurrence after treatment | At 12 months: CR 11% (2/18), PR 39% (7/18); mean time to response 3.5 months. |
| 40 | Middeldorp (2025) | Netherlands | Precancerous Cervical Lesions    CIN3+ | CRR 0.085 (95%CI 0.025, 0.24) (Cancer);  CRR 0.19 (95%CI 0.16, 0.23) (CIN3+) |
| 41 | Muresu et al. (2022) | Italy | HPV infection prevalence | HPV positivity: 48.1%.  Most prevalent genotypes:  HPV16 (36.3%),  HPV31 (18.7%),  HPV66 (11.9%),  HPV56 (11.4%),  HPV51 (10.7%).  HPV16 prevalence: vaccinated 18.3% vs unvaccinated 17.1% (no significant difference). |
| 42 | Moore et al. (2023) | Canada | HPV infection prevalence | No consistent pattern of protection against incident HPV in females.  No indication that recent vaccination reduced transmission in discordant couples or protected partners.  Results similar across vaccine-targeted, phylogenetically related, and unrelated HPV groups. |
| 43 | Na et al. (2024) | South Korea | HPV infection prevalence | HPV16/18 prevalence declined from 18.5% to 11.8% as vaccination rates increased (14.3% to 60.7%).  HPV16 VL reduced 6-fold by vaccination.  E2/E6 ratio increased: HPV16 1.4×, HPV18 5×. HPV16 persistent infection (>18 months) reduced from 31.0% to 21.6%. HPV18 infection (12–24 months) reduced from 35.5% to 21.1%.  Persistence rate: unvaccinated 90% vs vaccinated 70.6% for HPV16. Vaccination maintained episomal physical state for both HPV16/18. |
| 44 | Onuki et al. (2023) | Japan | HPV-related cancer | ICC incidence in women aged 20–29: declined from 3.6 to 2.8 per 100,000 women-years (2016–2019, p < .01); no decline in older age groups. 5Annual ICC cases aged 20–29: decreased from 256 to 135 (2011–2020, p < .0001).  HPV16/18 prevalence in ICC aged 20–29: declined from 90.5% to 64.7% (2017–2022, p = .05).  No similar trends in women ≥30y. |
| 45 | Palumbo (2025) | Italy | Precancerous Cervical Lesions    CIN2+  CIN3+ | p = 0.0100 for women aged ≥ 40    CIN1 treatment- positive HPV UV women=38%; V women = 18% (p = .017))    CIN2-3 treatment- positive HPV: UV women=18%, V women=8% (p=.035) |
| 46 | Pruski et al. (2023) | Poland | HPV infection prevalence | 76.5% cleared virus (p < 0.001)    HPV infection rate 9vVax vs unvax = 23.5% vs 88.9%, p < .001 |
| 47 | Pešut et al. (2024) | Croatia | HPV infection prevalence | Significant reduction only in HPV31  HPV31 (p = .02)  HPV16 (p=.731)  HPV18 (p=.318)  HPV45 (p=.892)  HPV58 (p=.186)  HPV33 (p=.501)  HPV52 (p=.611)  non-vaccine HPV types inc 11.9% (p=.02) |
| 48 | Pei et al. (2023) | United States | HPV-related cancer | Overall cervical cancer -0.7% (95%CI -1.1 to -0.3) Squamous cell carcinoma SCC -1.0 (95%CI -1.4 to -0.5)  Cervical adenocarcinoma AC +2.6% (95%CI 0.7 to 4.6) |
| 49 | Pruski et al. (2025) | Poland | HPV infection prevalence | 81.1% (V+LEEP) vs 57.1% (NV+LEEP)    Rate of HPV disappearance V=72.4% vs UV45.7% *(p* < .001*)* |
| 50 | Rasmussen (2020) | Denmark | HPV infection prevalence | Age annual percentage change AAPC:  Aged<20: -22.10% (95% CI: -35.27 to -6.26)  20–29 years (AAPC = −6.57, 95% CI: −10.63 to −2.33) |
| 51 | Rykkelid et al. (2024) | Norway | HPV infection prevalence | ARR: 12.1% (p=0.039) |
| 52 | Seong et al. (2020) | South Korea | HPV infection prevalence | Women vacinated >12mo prior aPR 0.35 (LSIL cohort)    Vaccinated ≥12mo prior [aPR]=0.51; 95% confidence interval [CI]=0.29–0.88)    Vaccinated individuals: lower proportion of HPV-16/18 (p=0.028) for three doses |
| 53 | Shing et al. (2021) | United States | Precancerous Cervical Lesions    CIN2+ | CIN2+ incident trends  AAPC -30.4 (95%CI): −35.4, −25.0) (MSA)  vs  -30.9 95%CI: −36.8, −24.5 (Rural) |
| 54 | Song et al. (2025) | South Korea | HPV infection prevalence | 27-39y persistent HPV infection (OR = 0.46; 95% CI: 0.22–0.96; p = 0.040).    progression to CIN2+ (HR = 0.38; 95% CI: 0.18–0.81; p = 0.011) |
| 55 | Silverberg (2020) | United States | HPV infection prevalence | RR: 0.81 (95% CI: 0.71–0.92) |
| 56 | Shiko et al. (2020) | Japan | Precancerous Cervical Lesions    CIN2+  CIN3+ | CIN2+ prevalence  RR: 0.24, 95%CI = 0.10 to 0.60;    CIN3+ prevalence  RR: 0.09, 95% CI:0.00–0.42(CIN3) |
| 57 | Tabibi et al. (2022) | United States | HPV-related cancer | % reduction in cancer incidence:  aged 15 to 24 years (−37.70% [95% CI, −42.24% to −32.75%]    aged 25 to 29 years (−16.16% [95% CI, −19.45% to −12.69%])    aged 30 to 39 years (−8.03% [95% CI, −9.90% to −6.12%] relative change *p* < .001    Cancer mortality:  aged 15 to 24 years (−43.35% [95% CI, −59.38% to −20.50%)    aged 25 to 29 years (relative difference, −47.71% [95% CI, −71.16% to −22.06%], relative change *p* = .001    aged 30 to 39 years (relative difference, −38.64% [95% CI, −55.64% to −15.25%], relative change *p* = .004 |
| 58 | Tozawa-Ono (2021) | Japan | Precancerous Cervical Lesions | significantly lower rate of:  ASC-US or worse (P < .0001),  ASC-H or worse (P < .0001)  LSIL or worse (P < .0001)  HSIL or worse (P < .0001) |
| 59 | Wen et al. (2024a) | Global | Precancerous Cervical Lesions | Precancerous lesions not related to non-infected HR-HPV types:  VE against HPV 16/18 = 82.7% (95% CI: 63.70-93.00%). (against non-infected)    Reinfection related to infected HR-HPV types:  VE against HPV16/18 not significant |
| 60 | Wen et al. (2024b) | China | HPV infection prevalence | 100% Risk Reduction |
| 61 | Zhao et al. (2022) | China | HPV infection prevalence | 100% efficacy (HPV 16/18 precancers)    median follow-up = 94 months  Trend of sustained protection ≤11 years |
